# Hemoglobin homeostasis in abdominal aortic aneurysm: diagnostic and prognostic potential of hemoglobin/heme and scavenger molecules

**DOI:** 10.1186/s12872-024-04131-3

**Published:** 2024-08-27

**Authors:** Sakshi Vats, Kristina Sundquist, Anton Grundberg, Jan Sundquist, Xiao Wang, Moncef Zarrouk, Anders Gottsäter, Ashfaque A Memon

**Affiliations:** 1 Center for Primary Health Care Research, Department of Clinical Sciences, Lund University, Malmö, Sweden; 2 University Clinic Primary Care Skåne, Region Skåne, Malmö, Sweden; 3 Department of Family and Community Medicine, McGovern Medical School, The University of Texas Health Science Center, Houston, Texas USA; 4 Vascular Centre, Department of Cardiothoracic and Vascular Surgery, Skåne University Hospital, Lund University, Malmö, S-205 02 Sweden; 5 Department of Medicine, Skåne University Hospital, Lund University, Malmö, S-205 02 Sweden; 6 Center for Primary Health Care Research, Wallenberg Laboratory, 5th floor, Inga Marie Nilsson’s gata 53, Malmö, 214 28 Sweden

**Keywords:** Abdominal aortic aneurysm, Hemoglobin homeostasis, Oxidative stress, Biomarker, Diagnostic potential, Prognostic potential

## Abstract

**Background:**

There is increasing evidence implicating hemoglobin/heme and their scavengers in oxidative stress-mediated pathologies, but information is limited in abdominal aortic aneurysm (AAA).

**Methods and results:**

In this case-control study, we assessed heme/heme-related markers in 142 men with AAA and 279 men with a normal aortic diameter consecutively recruited from an ultrasound screening program in Sweden. Enzyme-linked immunosorbent assays (ELISAs) were used to measure heme oxygenase-1 (HO-1) and hemopexin (Hpx) plasma levels, colorimetric assays for cell-free heme and whole blood hemoglobin (Hb) levels, and droplet digital PCR (ddPCR) and real-time PCR to determine haptoglobin (Hp) (pheno)type and genotype, respectively. Hpx and heme plasma levels at baseline were elevated, while HO-1 levels were lower in men with AAA (*p* < 0.001) and were significantly associated with AAA prevalence independently of potential confounders. A combination of heme and HO-1 showed the best diagnostic potential based on the area under the curve (AUC): 0.76, sensitivity: 80%, specificity: 48%. Additionally, when previously described inflammatory biomarker interleukin-6 (IL-6), was added to our model it significantly improved the diagnostic value (AUC: 0.87, sensitivity: 80%, specificity: 79%) compared to IL-6 alone (AUC: 0.73, sensitivity: 80%, specificity: 49%). Finally, Hb (positively) and Hpx (negatively) levels at baseline were associated with AAA growth rate (mm/year), and their combination showed the best prognostic value for discriminating fast and slow-growing AAA (AUC: 0.76, sensitivity: 80%, specificity: 62%).

**Conclusions:**

This study reports the distinct disruption of heme and related markers in both the development and progression of AAA, underscoring their potential in aiding risk stratification and therapeutic strategies.

**Supplementary Information:**

The online version contains supplementary material available at 10.1186/s12872-024-04131-3.

## Introduction

Abdominal aortic aneurysm (AAA) is a potentially lethal disease, predominantly affecting men over the age of 65 years [1, 2] with a global average prevalence of 1.46% (95% CI: 1.04–2.05) in males [2]. It is characterized by the irreversible weakening and dilatation of the abdominal aorta in the infrarenal region [3]. AAAs are most often asymptomatic and therefore not diagnosed until complications arise, incidentally, or through AAA screening programs [1]. The key diagnostic criteria for AAAs include a permanent, localized dilation of the infrarenal aortic diameter to ≥ 30 mm, as detected by imaging techniques like ultrasonography (US) or computed tomography (CT) scanning [1]. Although mainly asymptomatic, individuals with AAA face a lethal risk due to the continuous aneurysm growth that can lead to AAA rupture (rAAA) with a mortality rate of over 80% [4]. Consequently, several high-income countries, including Sweden, offer ultrasound screening for AAA to 65-year-old men, which has been shown to be cost-effective and associated with reduced AAA-related mortality [5–7]. According to current guidelines, individuals with small to medium asymptomatic AAAs (diameter < 55 mm in men) undergo regular US surveillance of their aortic diameter [1, 8] at intervals based upon baseline aortic diameter [8]. Surgical intervention is recommended for men with AAAs ≥ 55 mm [1, 8]. However, relying solely on aortic diameter as a prognostic tool for AAA has limitations [9, 10]. Therefore, there is an urgent need to identify diagnostic and prognostic biomarkers for AAA, which can potentially also serve as therapeutic agents or targets.

Oxidative stress is defined as an imbalance between toxic free radicals (reactive oxygen or nitrogen species) and antioxidants or protective mechanisms. Although there is increasing evidence implicating oxidative stress as a central player in the pathogenesis of AAA, the exact role and sources of oxidative stress and their potential as biomarker(s) of AAA diagnosis and prognosis remain unclear [11]. Hemoglobin (Hb) is a protein with a labile heme prosthetic group, found in red blood cells (RBCs) that carries oxygen throughout the body. When released from the RBCs, cell-free hemoglobin and resulting cell-free heme can lead to heme toxicity, which is regarded as both one of the sources as well as a consequence of oxidative stress and inflammation in various chronic diseases [12–14]. However, under physiological conditions, the presence of free hemoglobin and heme due to a low level of RBC lysis in the body induces the production of specific scavenger proteins that help to neutralize them [15]. One such scavenger protein is haptoglobin (Hp), which binds to free hemoglobin and forms a stable complex [14]. This complex prevents the harmful effects of free hemoglobin, including oxidative damage and inflammation [16]. Hemopexin (Hpx) is another circulating protein that binds and help neutralize free heme in circulation [16]. Upon successful binding by haptoglobin and hemopexin, free hemoglobin and heme are finally broken down through the induction of an acute phase enzyme, heme-oxygenase-1 (HO-1) [17]. Interestingly, Hp has three functionally different phenotypes: Hp1-1, Hp1-2 and Hp2-2, resulting from a well-known gene duplication [18]. Previous literature indicates towards differences between the three phenotypes, in terms of binding and clearance efficiency, as well as clinical consequences [19, 20]. The role of Hp for AAA occurrence and growth is not well established due to very few studies that had low sample sizes and used different low-throughput phenotyping methods [21–23]. Recently, a genetic polymorphism in the Hp gene (rs2000999) was identified as a strong predictor of circulating Hp levels [24]; however, it has not been studied in AAA. While there have been some studies examining Hp and Hpx separately in AAA, the results have been inconsistent. Additionally, no study so far has systematically investigated Hb, cell-free heme, Hp (phenotype and genotype) and plasma HO-1 in AAA presence and growth.

Heme toxicity can result from either a direct significant increase in free hemoglobin/heme in the circulation due to hemolysis or decline in their scavenger molecules [13]. Heme toxicity is especially important to study in the context of AAA because of its infrarenal location that is known to suffer from hemodynamic insults more often, leading to enhanced hemolysis and hence, free heme in the circulation [25, 26]. In the present study, we hypothesized that disruptions in hemoglobin/heme and their scavenger system play a role in AAA and its growth and could be exploited as potential biomarkers for diagnosis and prognosis of AAA. We therefore aimed to investigate the association(s) of whole blood hemoglobin, plasma (cell-free) heme, Hpx, HO-1, and Hp phenotype-genotype, with both occurrence and growth of AAA. As a secondary aim, we explored the diagnostic and potential of the above-mentioned markers and investigated whether they improve the discrimination potential of some already found markers.

## Materials and methods

All research practices were in accordance with the Declaration of Helsinki. This study was approved by the Ethics Committee of Lund University (2010/239) and all participants gave written informed consent prior to participation.

### Study population

Within an AAA ultrasound (US) screening program conducted at the Department of Vascular Diseases, Skåne University Hospital, all 65-year-old men from Malmö city and nearby municipalities are invited for screening [27]. The maximum infrarenal anteroposterior diameter of the aorta was measured using the leading-edge to leading-edge (LELE) method after US examinations by biomedical technicians or specially trained nurses using the LOGIQ E system from General Electric Healthcare Inc., United Kingdom. An aortic diameter of ≥ 30 mm was classified as AAA, while < 30 mm was categorized as normal. In 1% of the participants with inconclusive US examination, conventional CT scans (without contrast) were performed. During the period from 2010 to 2017, AAA prevalence among screened men was found to be 1.7% (415 out of 24,589), which aligns with the findings of a recent AAA screening study in Sweden^5^. Out of the total 415 AAA cases, 142 (34%) agreed to undergo physical examination, provide blood samples, and share their medical history. To establish a control (non-AAA) group, 279 men from the same cohort but with a normal aortic diameter (< 30 mm) were included. After the initial screening, regular ultrasound follow-ups were conducted for AAA patients, with the frequency determined by the size of the baseline aneurysm, following European recommendations and Swedish screening principles [1, 5]. As per the standard protocol, AAA patients with aortic diameter ≥ 55 mm were referred for surgery. As a result, follow-up data regarding AAA diameter was available for 113 out of 142 (80%) men with AAA. The AAA growth (mm/year) was calculated as: (latest aortic diameter – baseline diameter)/follow-up duration in years.

### Blood sampling and clinical assays

Fasting venous blood samples (6 mL) were taken from participants only at the time of enrollment using Ethylenediaminetetraacetic acid (EDTA) - containing collection tubes [Becton-Dickinson, Franklin Lakes, USA]. Collected blood samples were centrifuged at 4 °C at a speed of 1800 g for 15 min, and aliquots of plasma were flash frozen and stored at − 80 °C until later analysis. Routine clinical laboratory markers like whole blood Hb (g/L) were analyzed at the clinical chemistry department at Skåne University Hospital, Malmö, Sweden according to standard SWEDAC practice.

### Study colorimetric assays

Commercially available enzyme-linked immunosorbent assays (ELISAs) and guidelines were used to measure plasma levels of Hpx [ab108859, Abcam, USA] and HO-1 [ab207621, Abcam, USA]. Plasma heme was measured by a colorimetric assay following the manufacturer’s guidelines [DIHM-250, BioAssay Systems, Hayward, CA, USA]. The dilution factors used were: 1:400, 1:11, and 1:2 for Hpx, HO-1, and Heme assays, respectively. For Hpx and HO-1 ELISA assays, the absorbance was read at 450 nm and for the heme assay, 400 nm wavelength was used. The intra- and inter-assay coefficients of variation were 7.8% and 14.1% for Hpx; 3.9% and 11.3% for HO-1; and 7.5% and 9.6% for heme assays, respectively. Visible hemolyzed plasma samples (*n* = 6) were not utilized and plasma for 16 samples were not available/not enough for all current plasma-based assays. The plasma samples were analyzed in a random order to minimize bias. Plasma interleukin-6 (IL-6) was measured using commercially available ELISA assays (Pharmingen, San Diego, CA, USA) [28].

### Haptoglobin genotyping

#### Hp typing

The haptoglobin protein is expressed as three different individual-specific phenotypes (Hp1-1, Hp1-2, and Hp2-2) differing in size/molecular weight, based on the expression of two codominant genetic alleles (Hp1 and Hp2). In the present study, a new Droplet digital PCR (ddPCR) assay was developed for the determination of three Hp types in whole blood DNA samples, that arise due to presence of a 1.7 Kb gene duplication in Hp gene. The Hp2 allele or duplicated Hp gene region (Hg19 chr16:72058115 to 72058237) was quantified as the target and another stable gene region (always two copies in the diploid genome) was used as the reference during the ddPCR reaction. The protocol is described in detail below.

Absolute DNA was extracted from whole blood samples using the QIAamp^®^ DNA Blood kit and manufacturer’s guidelines [Qiagen, U.S.A.]. DNA concentration was determined using Thermo Scientific™ NanoDrop 2000 and all samples were normalized to 0.5ng/µL before the run. Analysis on 12 individuals was not possible due to insufficient or unavailable DNA samples. For absolute target duplication quantification, primer pair and probe targeting the duplicated region in Hp2 allele (HP2) was used. Primer pair and probe targeting the eukaryotic translation initiation factor 2 C-1 (EIF2C1) were used for reference quantification. Information regarding the used primers and probes can be availed from www.bio-rad.com using these IDs: HP2 (assay ID: dHsaCNS626037518; context sequence: GCCAGAGAGTTTGCTATTTGGAA ATTGTTCCCAGTGAACCGTGAAAAGTCAGATGAGCGGGAGCTGCTCTGCACATCAATCTCCTTCCACCCCGAATAGAAGCTCGCGAACTGTATTATTTTT) and EIF2C1 (assay ID: dHsaCP1000002; context sequence: TGGTTCGGCTTTCACCAGTCTGTGCGCCCTGCCATGTGGAAGATGATGCTCA ACATTGATGGTGAGTGGGGAGAGCTATGGAGCCAGGGGCACCCCAAGTCCAGTGACCACACTCCCAGCCTC). FAM fluorophore was used in conjugation with probes targeting HP2 and HEX-conjugated probes were used for targeting the reference, EIF2C1. The ddPCR amplification was performed with a total multiplex reaction volume of 22 µl with 3ng of input DNA, 0.9X primer-probe concentrations, HaeIII (5U/reaction) [Thermo Scientific, USA], and 1X of ddPCR supermix for probes. The input DNA amount of 3ng was arrived at after testing multiple DNA concentrations for target and reference assays alone or in combination. DdPCR plates were incubated for 30 min at room temperature to provide optimal time for restriction digestion by HaeIII. Following incubation, samples underwent fractioning (∼20,000 droplets, and theoretically 1 DNA molecule per droplet) by an automated droplet generator from the QX200 Droplet Digital PCR system [Bio-Rad, Hercules, CA]. DdPCR temperature cycling steps were as follows: single cycle of enzyme activation at 95° C for 10 min, followed by 40 cycles of denaturation (94° C for 30 s) and annealing (60° C for 1 min) and ultimately one final 10-minute cycle of enzyme deactivation at 98° C. For maximum droplet recovery (∼20,000 droplets), the ddPCR plate was stored at 4° C overnight, prior to final fluorescence reading by the QX200 Droplet Digital PCR system plate reader [Bio‐Rad, Hercules, CA]. The absolute copy number of target (HP2) and reference (EIF2C1) in units of copies/µL was determined by fitting the fraction of respective positive droplets to a Poisson distribution using the QuantaSoft™ Software. For determination of the three Hp types, the ratio of absolute copies of target (HP2) to reference (EIF2C1) was estimated. As EIF2C1 always has two copies in the diploid human genome, ratios of 1 represented two copies of the target Hp2 allele (Hp2-2), 0.5 represented a heterozygous state (Hp1-2), and 0 represented absence of Hp2 allele (Hp1-1). The intra and inter assay coefficient of variation for the hp typing ddPCR assay were: 7.08% and 8.12%, respectively.

#### Hp rs2000999 genotyping

Pre-designed TaqMan^®^ SNP Genotyping Assay (SNP ID: rs2000999, assay ID: C__11439054_10) with VIC and FAM fluorescent probes targeting NC_000016.10:g.72074194G > A polymorphism was used for genotyping on whole blood DNA samples. Real-time PCR run was performed on the Bio-Rad CFX384 real-time PCR system [Bio-Rad, Hercules, CA] according to manufacturer’s guidelines [Thermo Scientific, USA]. For allelic discrimination analysis to determine individual genotypes, Bio-Rad CFX manager software was used [Bio‐Rad, Hercules, CA].

### Statistical analysis

RStudio version 2022.02.3 was used to perform all the statistical analyses [29]. Student’s t-test and Mann-Whitney test were used to test differences in normally and non-normally distributed continuous data, respectively. Pearson’s chi-square (χ2) test was used for categorical variables. Continuous variables were expressed as the mean and standard deviation (SD), or median (IQR) and categorical variables were presented as count and percentages. The main study exposures had less than 10% missing data which were imputed using the “MissForest” [30] method based on random forest algorithms that are considered highly suitable for imputation of missing laboratory data [31]. To account for missing values in the covariates, a missing-indicator approach was used. Sensitivity analysis without imputation or complete case analysis (CCA) were also performed (Table S8-10). To test the association between the outcomes (AAA vs. non-AAA, and fast vs. slow growing AAA) and study exposures, logistic regression (Uni and multi-variable) analysis was performed. An extreme growth rate value (48 mm/year) was excluded (final n = 112). AAAs with growth rate < 2.5 mm/year were classified as slow growing and ≥ 2.5 mm/year were categorized as fast growing, based on average growth rate in small-medium AAAs from previous studies [32, 33]. Logistic regression analysis between AAA prevalence and study markers was adjusted for as follows: model a (cardiovascular disease [CVD], smoking pack-years, usage of metformin, antihypertensive and lipid-lowering medication); supplementary model b (smoking pack-years, diabetes, cancer, IL-6, BMI, cholesterol, and high-density lipoprotein [HDL]). Adjustment with baseline aortic diameter, IL-6, body mass index (BMI), and metformin use was done while testing the linear association between the study exposures and AAA growth. The correlations between aortic diameter/AAA growth rate and study markers were evaluated using the Spearman’s correlation coefficient test. To test the associations between the study markers and natural logarithm (ln) transformed AAA growth rate, linear regression analysis was performed. To assess the diagnostic (AAA vs non-AAA) and prognostic (fast vs slow growing AAA) potential of the study markers, receiver-operating characteristic (ROC) curve analysis was used. Additionally, to determine the discriminatory power of individual or combined markers, various diagnostic/prognostic parameters including sensitivity, specificity, and area under the ROC curve (AUC) with a 95% confidence interval (CI) were also estimated. To compare the discriminatory performance of two or more correlated ROC curves, Delong’s test for correlated ROCs was performed. P-value < 0.05 was considered significant.

## Results

### Baseline study population characteristics

The present study had a total cohort size of 421 men, 142 with and 279 without AAA. The distribution of baseline clinical features and other study variables amongst men with and without AAA and the overall study population is presented in Table 1. The median aneurysm growth rate in the AAA group was 1.7 mm/year. Men with AAA had higher smoking pack-years, BMI, plasma IL-6, plasma creatinine, and triglyceride levels than men without AAA. Levels of total-, HDL-, and LDL-cholesterol were lower in the AAA group as compared to men with normal aorta. Men with AAA had a higher lipid-lowering and antihypertensive medication usage, as well as concurrent CVD and type 2 diabetes, compared to men without AAA. Baseline clinical characteristics for individuals in the present study without missing study variable data are also presented (Table S1).

**Table 1:** Baseline clinical characteristics of 65-year-old men with and without abdominal aortic aneurysm (AAA) at ultrasound screening

Variable	Total *n* = 421	Without AAA *n* = 279	With AAA *n* = 142	*p*-value
Baseline aortic diameter (mm), mean (SD)	25.7 (10.1)	19.3 (2.4)	38.2 (7.5)	< 0.001^A^
Growth rate (mm/year), median (IQR) Missing, n (%)	1.7 (2.3)	-	1.7 (2.3) 30 (21.1)	-
Systolic BP (mmHg), mean (SD)	145.0 (17.7)	146.0 (18.1)	143.0 (16.8)	0.10^A^
Diastolic BP (mmHg), mean (SD)	85.7 (9.6)	85.9 (9.8)	85.5 (9.3)	0.71^A^
p-creatinine (µmol/L), mean (SD) Missing, n (%)	86.8 (18.1) 14 (3.3)	85.0 (13.5) 6 (2.2)	90.4 (24.6) 8 (5.6)	0.018^A^
p-cholesterol (mmol/L), mean (SD) Missing, n (%)	5.1 (1.2) 14 (3.3)	5.3 (1.2) 6 (2.1)	4.7 (1.2) 8 (5.6)	< 0.001^A^
p-triglycerides (mmol/L), median (IQR) Missing, n (%)	1.5 (1.0) 26 (6.2)	1.4 (1.0) 13 (4.7)	1.6 (1.0) 13 (9.1)	0.002^B^
p-HDL (mmol/l), mean (SD) Missing, n (%)	1.3 (0.4) 14 (3.3)	1.4 (0.4) 6 (2.2)	1.2 (0.3) 8 (5.6)	< 0.001^A^
p-LDL (mmol/l), mean (SD) Missing, n (%)	3.3 (1.1) 23 (5.5)	3.5 (1.0) 14 (5.0)	3.0 (1.1) 9 (6.3)	< 0.001^A^
p-glucose (mmol/l), mean (SD) Missing, n (%)	6.3 (2.4) 18 (4.3)	6.2 (2.3) 9 (3.2)	6.6 (2.4) 9 (6.3)	0.16^A^
p-IL-6 (pg/mL), median (IQR) Missing, n (%)	1.9 (2.3) 84 (20)	1.5 (1.6) 51 (18.3)	3.2 (4.3) 33 (23.2)	< 0.001^B^
BMI (kg/m^2^), mean (SD) Missing, n (%)	27.5 (4) 55 (13.1)	27 (3.9) 28 (10)	28.4 (4.2) 27 (19)	0.003^A^
Metformin, n (%) Missing, n (%)	28 (6.7) 64 (15.2)	13 (4.7) 41 (14.7)	15 (10.6) 23 (16.2)	0.018^C^
Lipid-lowering drug use, n (%) Missing, n (%)	149 (35.4) 64 (15.2)	77 (27.6) 41 (14.7)	72 (50.7) 23 (16.2)	< 0.001^C^
Anti-hypertensive drug use, n (%) Missing, n (%)	200 (47.5) 64 (15.2)	111 (39.8) 41 (14.7)	89 (62.7) 23 (16.2)	< 0.001^C^
AAA family history, n (%) Missing, n (%)	27 (6.4) 154 (36.6)	13 (4.7) 145 (52.0)	14 (9.9) 9 (6.3)	0.82^C^
CVD, n (%) Missing, n (%)	93 (22.1) 6 (1.4)	41 (14.7) 0 (0)	52 (36.6) 6 (4.2)	< 0.001^C^
Type 2 diabetes, n (%) Missing, n (%)	32 (7.6) 6 (1.4)	15 (5.4) 0 (0)	17 (12.0) 6 (4.2)	0.011^C^
Cancer, n (%) Missing, n (%)	52 (10.4) 6 (1.4)	29 (10.4) 0 (0)	23 (16.2) 6 (4.2)	0.060^C^
Smoking ≥ 15 pack-years, n (%) Missing, n (%)	159 (37.8) 68 (16.2)	72 (25.8) 35 (12.6)	87 (61.3) 33 (23.2)	< 0.001^C^

HDL: high density lipoprotein, LDL: low density lipoprotein, Hb: Hemoglobin, CVD: cardiovascular disease, IL-6: Interleukin-6, BMI: Body mass index, p: plasma

Data are represented as mean (SD), median (IQR), or count (percentage). A = Student’s t-test; B = Mann Whitney test; C = χ2 test

### Associations of heme-related markers with AAA prevalence

#### Levels in AAA and non-AAA and association with AAA risk

**Fig. 1:**
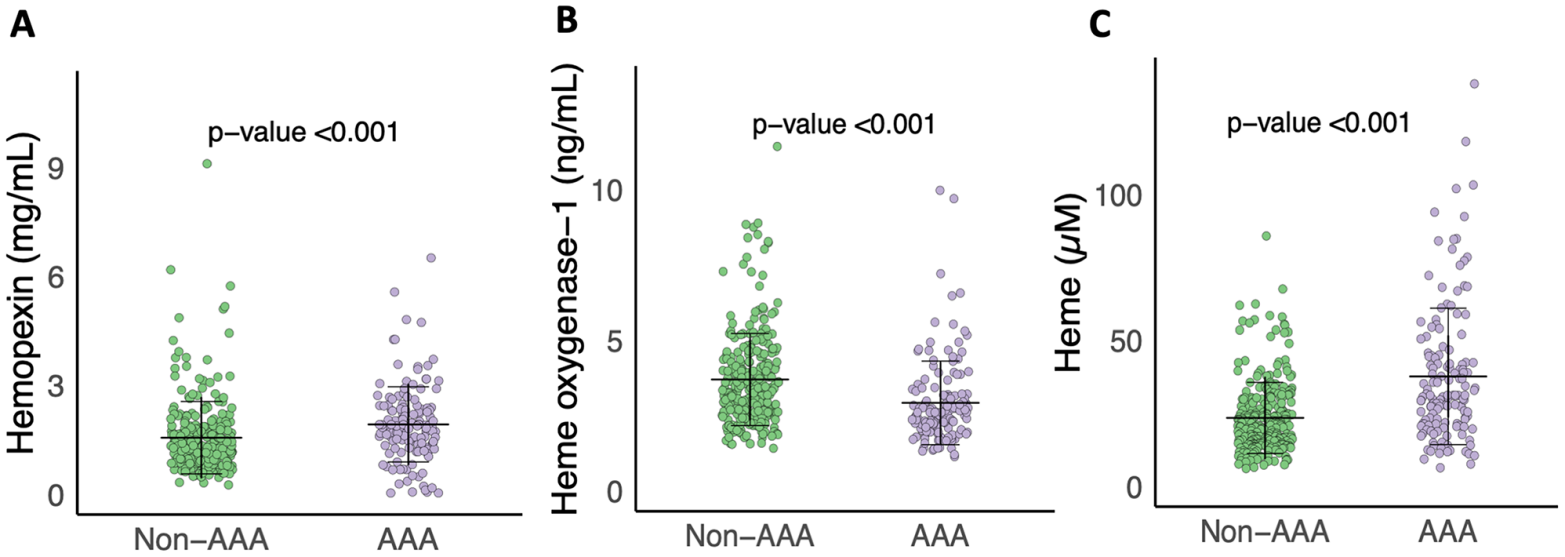
Plasma concentrations of **(A)** Hemopexin, **(B)** Heme-oxygenase-1 (HO-1) and **(C)** Heme, between AAA and non-AAA groups. The horizontal (black) lines denote the mean value and error bars represent the standard deviation (SD) in each group. P-value determined by Student’s t-test on standardized marker values

Plasma levels of Hpx (mean: 1.94 vs. 1.56 mg/mL) and heme (mean: 37.69 vs. 23.46 µM) were significantly elevated in AAA patients as compared to individuals without AAA at screening (*p* < 0.001) (Table 2; Fig. 1). Conversely, individuals with AAA exhibited significantly lower levels of plasma HO-1 (mean: 2.92 vs. 3.70 ng/mL) than the non-AAA group (*p* < 0.001) (Table 2; Fig. 1). These associations remained statistically significant even after adjusting for potential confounding factors (Table 2 and S11). Furthermore, plasma Hpx and heme showed a positive linear correlation, while plasma HO-1 correlated negatively with aortic diameter at screening in the overall study population (*p* < 0.01, Spearman’s correlation, Table S3.). However, when sub-grouped into AAA and non-AAA, no significant linear relationship was found (Table S3). Additionally, both Hp type (Hp1-1, Hp1-2, and Hp2-2) and genotype (rs2000999), showed no association with the occurrence of AAA (Table 2) or aortic diameter at screening (Table S2).

In terms of AAA risk, the per unit increase in standardized (mean = 0 and SD = 1) plasma values of Hpx and heme were associated with 1.42- and 2.48-times higher odds of having AAA at screening, respectively (Table 2 and S11). On the other hand, the per unit increase in standardized HO-1 plasma levels were associated with lower odds of having AAA at screening (OR (95%CI): 0.51(0.38–0.66)) (Table 2 and S11).

**Table 2:** Levels of heme-related markers in Non-AAA and AAA and their association with AAA prevalence (odds ratio (OR)) and diagnostic potential for AAA (area under curve (AUC))

Marker	Non-AAA	AAA	*p*-value	Adjusted *p*-value^a^	OR^1^	95% CI^1^	AUC^2^	95% CI^2^	*p*-value^2^
Hpx (mg/mL), mean (SD)	1.56 (1.0)	1.94 (1.03)	< 0.001	< 0.001	1.42	1.16–1.77	0.66	0.60–0.72	< 0.001
HO-1 (ng/mL), mean (SD)	3.70 (1.53)	2.92 (1.39)	< 0.001	< 0.001	0.51	0.38–0.66	0.68	0.63–0.74	< 0.001
Heme (µM), mean (SD)	23.46 (12.2)	37.69 (23.4)	< 0.001	< 0.001	2.48	1.92–3.28	0.71	0.66–0.77	< 0.001
Hb (g/L), mean (SD)	148.1 (10.1)	147.0 (11.2)	0.31	0.45	0.90	0.73–1.10	0.54	0.48–0.60	0.10
Hp type, n (%) Hp1-1 Hp2-1 Hp2-2	50 (17.9) 136 (48.8) 93 (33.3)	23 (16.2) 68 (47.9) 50 (35.9)	0.83	0.73	0.92 Ref 1.10	0.51–1.62 - 0.70–1.72	0.52	0.46–0.57	0.27
Hp genotype, n (%) GG GA AA	177 (63.4) 92 (33.0) 10 (3.6)	85 (59.9) 49 (34.5) 8 (5.6)	0.55	0.85	Ref 1.11 1.67	- 0.72–1.71 0.62–4.37	0.52	0.47–0.57	0.20

Hpx: Hemopexin; HO-1: Heme-oxygenase-1; Hb: Hemoglobin; Hp: Haptoglobin.^a^ Model a: Adjusted for smoking ≥ 15 pack-years, CVD, and medication (hypertension, lipid, metformin) Model b presented in Table S11. ^1^ Odds ratios and 95% CI for standardized (continuous) biomarkers (mean = 0 and SD = 1). ^2^ AUC, 95% CI, and p-value for AUC calculated using ROC analysis with AAA v/s non-AAA as outcome

#### Diagnostic potential of identified heme-related markers for AAA

**Fig. 2:**
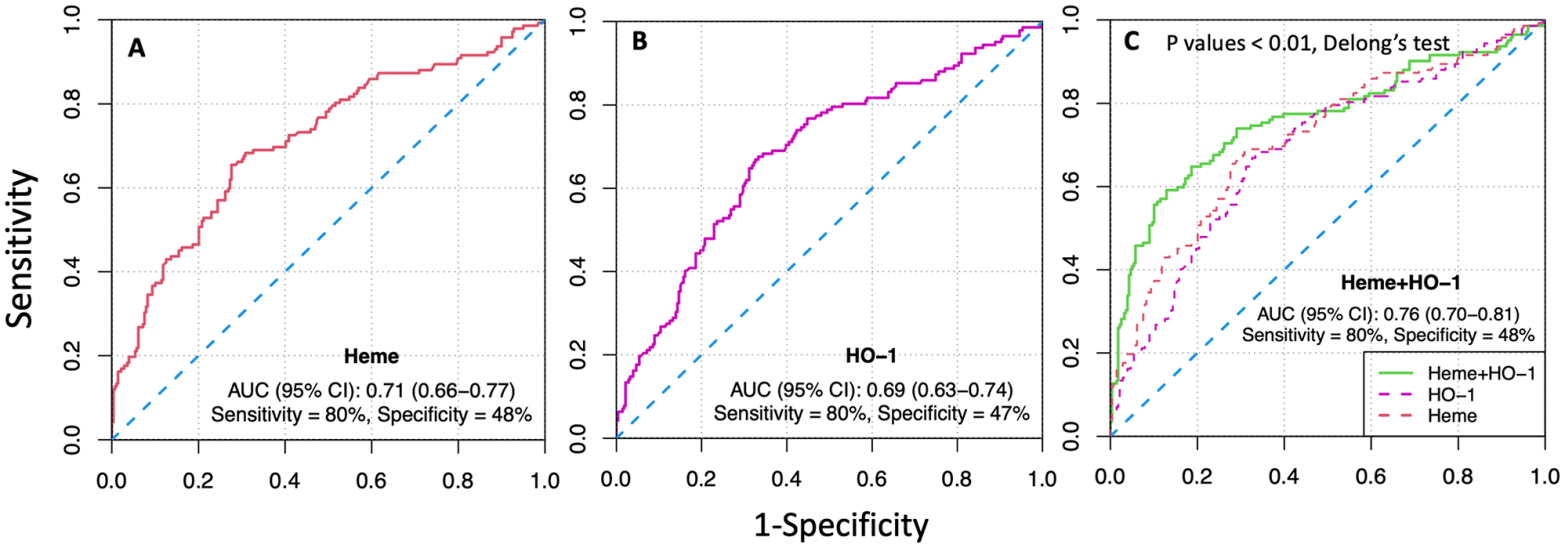
Receiver operating characteristic (ROC) curves showing sensitivity, 1-specificity, area under curve (AUC) scores, and 95% CI values for **(A)** Heme, **(B)** Heme-oxygenase-1 (HO-1), and **(C)** for combined Heme and HO-1 as best diagnostic model in the current study for discrimination between men with and without AAA. Y-axis presents sensitivity range and X-axis denotes (1-Specificity). P values: p-values estimated by Delong’s test for correlated ROCs (Heme + HO-1 vs. Heme or HO-1)

To evaluate the diagnostic value of the candidate markers, we performed ROC curve analysis. The area under the curve (AUC) values ,95% CI and p-values of the candidate markers are presented in Table 2. Among individual studied markers, heme showed the best diagnostic potential based on AUC (AUC, 0.71; 95% CI 0.66–0.77). It demonstrated a sensitivity of 80% and a specificity of 48%, as illustrated in Fig. 2(A). Heme was followed by HO-1, with an AUC (95% CI) of 0.69 (0.63–0.74), and sensitivity and specificity of 80% and 47%, respectively (Fig. 2(B)). Heme and HO-1 showed similar discrimination as they did not differ significantly (p-value = 0.43) when compared using Delong’s test for correlated ROCs, Table S3. Interestingly, the combination of heme and HO-1 exhibited the best discriminatory ability with an AUC (95% CI) of 0.76 (0.70–0.81), and sensitivity and specificity of 80% and 48%, respectively (Fig. 2C, Table S4). This combination significantly improved the discriminatory ability compared to the individual markers HO-1 and heme (p-values < 0.01, Delong’s test for correlated ROCs, Table S3).

### Associations of heme-related markers with AAA growth

#### Levels with respect to AAA growth

**Fig. 3:**
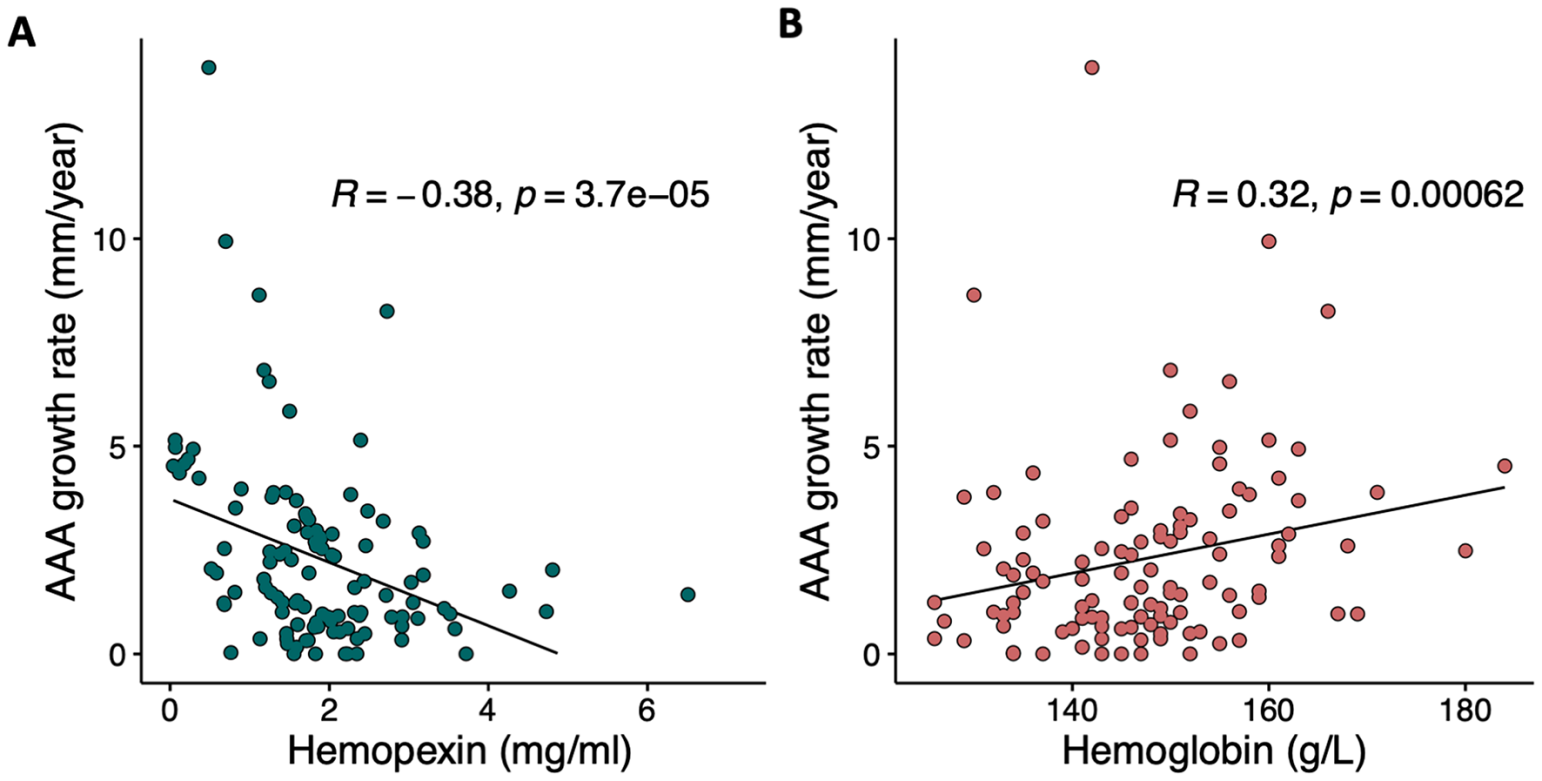
Linear association of AAA growth rate (mm/year) in 65-year-old men with **(A)** Plasma hemopexin (Hpx, mg/mL) and **(B)** with whole blood hemoglobin (g/L). The plots display linear regression lines, Spearman’s (rho) correlation coefficients (R) and p-values (*p*) from Spearman correlation analyses

Hpx exhibited a significant inverse linear association with AAA growth rate, as indicated by Spearman’s correlation coefficient (rho = -0.38, *p* < 0.001). On the other hand, whole blood hemoglobin showed a positive association with growth rate (Spearman’s rho = 0.32, *p* < 0.001) (Fig. 3, Table S4). To further investigate these associations, linear regression analyses were performed between log-transformed AAA growth rate and standardized study markers (Table 3). Even after adjusting for baseline aortic diameter, BMI, IL-6, and metformin use, the inverse relationship between Hpx and growth rate remained significant (adjusted beta = -0.17, *p* = 0.003). Similarly, the positive association between Hb and growth rate also remained significant (adjusted beta = 0.13, *p* = 0.03) (Table 3).

**Table 3:** Associations between biomarkers and AAA growth as continuous variable

Biomarker	β	*p*-value	Adjusted β^1^	*p*-value^1^	AUC^2^	95% CI^2^
Hpx	-0.19	< 0.001	-0.17	0.003	0.68	0.57–0.78
HO-1	0.12	0.19	-0.009	0.42	0.58	0.47–0.69
Heme	0.07	0.13	0.06	0.28	0.70	0.60–0.80
Hb	0.16	0.002	0.13	0.03	0.68	0.57–0.79
Hp (phenotype) Hp1-1 Hp2-1 Hp2-2	0.10 Ref 0.00	0.53 - 0.99	0.11 Ref 0.10	0.49 - 0.49	0.57	0.46–0.67
Hp (genotype) GG GA AA	Ref -0.03 0.07	- 0.80 0.76	Ref -0.06 -0.27	- 0.63 0.43	0.52	0.42–0.62

Marker values were standardized, and AAA growth rate was natural logarithm (ln) transformed

Hpx: Hemopexin; HO-1: Heme-oxygenase-1; Hb: Hemoglobin; Hp: Haptoglobin; IL-6: interleukin-6

^1^ Adjusted for baseline diameter, IL-6, BMI and Metformin use

^2^ AUC and 95% CI calculated using ROC analysis with fast v/s slow growing AAA as outcome

#### Prognostic potential of identified heme-related markers for AAA growth

**Fig. 4:**
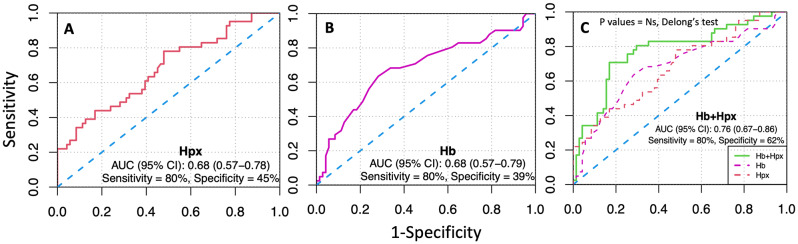
Receiver operating characteristic (ROC) curves showing sensitivity, 1-specificity, area under curve (AUC) scores, and 95% CI values for **(A)** Hemopexin (Hpx), **(B)** Hemoglobin (Hb), and **(C)** for combined Hpx and Hb as prognostic models in the current study for discrimination of individuals with slow and fast-growing AAAs. Y-axis presents sensitivity range and X-axis denotes (1-Specificity). P values: p-values estimated by Delong’s test for correlated ROCs. (Hb + Hpx vs. Hb or Hpx). Ns: non-significant (p-value > 0.05)

To assess the prognostic value of the candidate markers, we conducted ROC curve analysis and obtained the corresponding area under the curve (AUC) values, 95% confidence intervals (CI), and p-values, as presented in Table 3. Among all investigated markers, Hpx exhibited the best diagnostic potential based on AUC of 0.68 with a sensitivity of 80% and specificity of 45% (Fig. 4A), followed by Hb (AUC; 0.68, sensitivity = 80% and specificity = 39%) (Fig. 4B) (Table 3, S5). Furthermore, when we combined Hb and Hpx, we observed an improved model that outperformed either biomarker alone (Fig. 4C and Table S5). The combined model had an AUC of 0.76 with a sensitivity of 80% and specificity of 62%. However, it is important to note that the difference in discriminatory potential between the combined model and individual Hb and Hpx markers did not reach statistical significance (p-value = 0.07 and 0.06, respectively, as determined by Delong’s test for correlated ROCs).

### Evaluation of improvement in AAA discrimination of previously published markers with the current heme-related markers

**Fig. 5:**
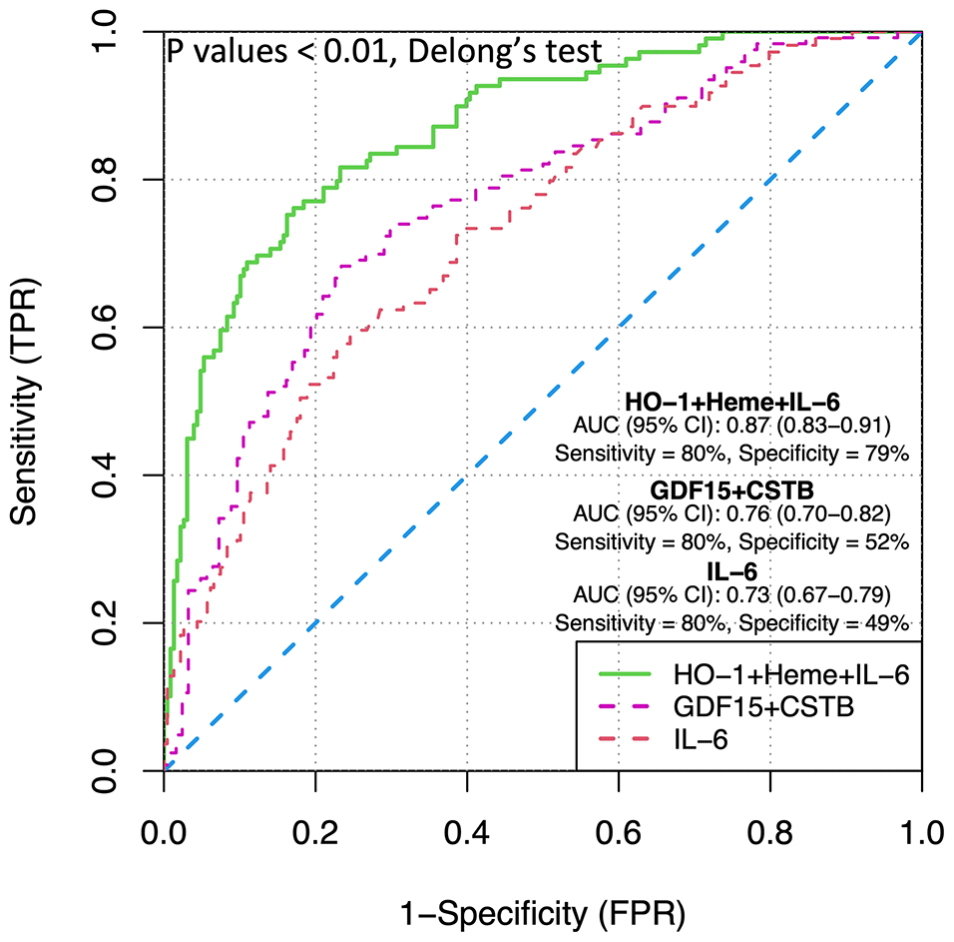
Receiver operating characteristic (ROC) curves showing sensitivity, 1-specificity, area under curve (AUC) scores, and 95% CI values for IL-6 (red curve), combination of GDF15-CSTB (pink curve), and combination of HO-1 + heme + IL-6 (green curve) as diagnostic models for discrimination of individuals with and without AAA. Y-axis presents sensitivity range and X-axis denotes (1-Specificity). P values: p-values estimated by Delong’s test for correlated ROCs (HO-1 + heme + IL-6 vs. GDF15 + CSTB or IL-6). GDF-15: growth differentiation factor-15; CSTB: cystatin B; IL-6: interleukin-6

In a previous study conducted on the current study population (*n* = 268), the combination of growth differentiation factor-15 (GDF-15) and cystatin B (CSTB) showed the best diagnostic potential (AUC (95% CI); 0.76 (0.70–0.82), sensitivity = 80% and specificity = 52%) (Fig. 5) [34]. The well-known inflammatory marker IL-6 has also been positively associated with AAA prevalence, and showed potential as a diagnostic marker in this study population (AUC (95% CI); 0.73 (0.67–0.79), sensitivity = 80% and specificity = 49%) (Fig. 5) [35]. We also checked multiple combinations of the markers investigated in this study (significantly differing between men with and without AAA) with GDF15, CSTB, and IL-6. Our results showed that combining heme and HO-1 with IL-6 had the best diagnostic potential for AAA (AUC; 0.87, 95% CI 0.83–0.91), with a sensitivity of 80% and specificity of 79% leading to a significant improvement of the previous models with GDF15 + CSTB and IL-6 alone. (p-value *< 0.01*, Delong’s test for correlated ROCs, Fig. 5 and Table S6.). No similar improvement was observed in the models of prognosis or AAA growth rate (Table S7).

## Discussion

Excess of extracellular hemoglobin-heme are currently defining a new model of heme toxicity that is implicated in the pathogenesis of a growing number of chronic, oxidative stress-related diseases [12]. Considering this, in the present study, we aimed to investigate the association between free total heme, hemopexin (Hpx), and heme-oxygenase-1 (HO-1), as well as hemoglobin (Hb) and haptoglobin (Hp) phenotype-genotype, and AAA and its growth rate. Our results indicate towards a general and distinct disruption in these heme/or heme-related proteins relevant for both AAA prevalence and growth in men.

Men with AAA had significantly higher levels of plasma heme and Hpx, but lower levels of HO-1 at baseline. Furthermore, among the current study markers, the combination of heme and HO-1 showed the best diagnostic potential to discriminate between AAA and non-AAA individuals. Increased plasma heme has previously been associated with vascular conditions, like atherosclerosis and preeclampsia [36]. To the best of our knowledge, there is no study investigating the role of plasma Hpx and HO- 1 in AAA occurrence. There are three main mechanisms by which excess of cell-free heme can lead to or exacerbate vascular pathologies, like AAA. Firstly, free heme is a highly pro-oxidant molecule, which leads to direct oxidative damage of lipids membranes, nucleic acids, and protein degradation. Secondly, free heme is also hemolytic in nature, i.e., it can lead to further red blood cell instability, lysis, and anemia, forming a vicious loop. Anemia has previously been associated with adverse treatment outcomes in AAA [37]. Lastly, heme is a strong pro-inflammatory stimulus, which can induce migration of neutrophils, platelets, and other circulating cells to the vascular endothelium, aggravating the inflammation and thrombosis at the affected site [13]. Overall, consequences of heme toxicity align well with pathophysiological aspects of AAA, but the association between plasma heme and AAA had not been previously investigated. However, a study in AAA has reported increased accumulation of iron in aortic wall tissue, which we believe could be a result of an excess of cell-free Hb or heme in the circulation [38, 39]. Another study reported an increase in abdominal aortic stiffness in hemolysis-driven, beta-thalassemia major patients which correlated with liver iron concentration [40]. Ferritin, a well-established marker for assessing overall body iron stores also in older men, is not associated with AAA presence, size and growth [41, 42]. However, this assay does not account for the iron sequestered in heme and hemoglobin molecules, underscoring the importance of assessing these markers in the context of AAA.

The role of hemopexin has been largely unclear in AAA, mainly due to inconsistency in previous findings [43, 44]. A previous study that compared serum proteins in 20 AAA and 20 non-AAA individuals using 2D gel electrophoresis, reported downregulation of Hpx levels. One thing to note here is that, unlike our study, the majority of cases in the study by Spadaccio and colleagues had large AAAs (mean aortic diameter = 78 ± 12 mm) and had been admitted to hospital for surgery [44]. Another study in AAA patients with a maximum aneurysm diameter of 50 mm, using mass spectrometry analysis, observed a similar upregulation of Hpx in AAA patients at baseline [43], in line with our findings. These observations suggest that the role of Hpx might differ in different stages of AAA development. Therefore, it is possible that increases in circulating Hpx levels observed in the current study could be a marker of earlier stages of AAA.

We observed lower plasma HO-1 levels in individuals with AAA as compared to those without. This finding is in line with the biological theory of a protective, anti-oxidative and anti-inflammatory role of HO-1 [45]. A previous study of AAA on animal models has shown that HO-1 deficiency increases the incidence of AAA and AAA rupture that is accompanied by increased reactive oxygen species (ROS) levels, vascular smooth muscle cell apoptosis and inflammation [46]. Another in vivo study on AAA reported a dual role of HO-1, where it protected from future incidence of AAA but enhanced the severity of already formed AAA [47]. Another study observed increased levels of serum HO-1 in patients undergoing elective repair or with already ruptured AAA. However, it is important to note that patients with venous vessel varicose were treated as the control group in that study, making the findings difficult to interpret. In line with our findings, plasma levels of HO-1 were found to be significantly reduced in individuals with peripheral artery disease (PAD) as compared to non-PAD individuals at baseline [48]. Taken together, more longitudinal studies are needed to infer causality or to understand the dynamics of HO-1 in AAA.

Interestingly, unlike the positive association with AAA prevalence, we observed a significant negative correlation between plasma Hpx and AAA growth rate. This finding is in line with previous studies accounting the highly cardio-protective role of Hpx, especially in alleviating effects of excess heme [49, 50]. We speculate that the reason behind this interesting observation.

in our study is a context specific role of Hpx. As discussed above, Hpx in serum was downregulated in patients with large AAAs while upregulated in patients with small to medium AAAs, possibly representing differences in associations with late and early stage of AAA [43, 44]. It is possible that Hpx is upregulated in the early stage of the disease as a compensatory mechanism, but that the magnitude of upregulation/compensation is associated with better prognosis i.e., the higher the Hpx level at baseline, the slower the growth rate. More longitudinal data are needed to confirm this hypothesis.

A notable finding in the current study was the association between high whole blood Hb levels at baseline and higher AAA growth rate, independent of baseline aortic diameter. This observation is supported by other studies reporting a positive association between Hb levels and CVD incidence, chronic obstructive pulmonary disease (COPD)- and all-cause mortality, as well as development of the metabolic syndrome [51–53]. Interestingly, a U-shaped association between Hb and CVD incidence or outcome has been observed, meaning both anemia and high Hb levels are implicated [51, 52]. As we did not have anemic individuals in the current study population (3% individuals had Hb < 130 g/L and only 2 individuals had Hb < 120 g/L), we could not observe a U-shaped association. In terms of potential biological mechanism, high levels of Hb are associated with increased blood viscosity leading to decreased blood flow and perfusion to tissues [54]. In addition, it can also lead to platelet activation, promoting thrombosis and oxidative stress, potentially contributing directly to AAA pathophysiology [55]. In the current study, combining Hpx and Hb showed a good prognostic value for discrimination between fast and slow growing AAAs (AUC = 77%, Sensitivity = 80% and Specificity = 62%). A possible explanation behind the association between high baseline hemoglobin levels and low baseline hemopexin with aneurysm growth rate (but not AAA prevalence), could be a steady conversion of RBC bound Hb to toxic cell-free Hb/heme through gradually increasing hemolysis and decline in Hb scavenging capacity, along the AAA disease progression.

Of note, we did not observe any association between Hp phenotype- and circulating level-predicting genotype (rs2000999), neither with AAA prevalence nor growth. Our results add to the existing contradictory evidence on the role of Hp phenotype and genotype in AAA and CVD [21–23, 56]. However, unlike, the previous studies, we used highthroughput and robust methods for Hp typing as well as genotyping in a relatively large study population. It is worth mentioning that there is evidence supporting the role of circulating Hp levels in AAA without accounting for the genotype or phenotype [57]. It is, therefore, possible that the role of Hp in AAA is independent of phenotype and the currently studied functional genotype, respectively, but this hypothesis requires further validation.

Overall, the heme homeostasis-related markers investigated in the current study, showed significant and distinct associations with AAA prevalence and growth. They further showed a potential of diagnosis and prognosis of AAA, in combination as well as individually. In fact, adding heme and HO-1 to the well-known inflammatory marker IL-6 improved the diagnostic potential compared to the previously described biomarker combination (GDF15 + CSTB) and IL-6 alone within the same study cohort [34, 35]. The enhanced diagnostic potential observed with the combination of IL-6, heme, and HO-1 might reflect the integration of both oxidative stress and inflammatory aspects in the pathophysiology of AAA. There is accumulating evidence supporting the potential of hemoglobin/heme and their scavengers as biomarkers in disease occurrence and severity [36, 58] and there is an ongoing effort to exploit the therapeutic potential of these scavenger molecules in other pathologies [59]. These observations further support our current findings and suggest potential clinical applicability of these biomarkers for aiding diagnosis and prognosis in AAA, and potentially even for future therapeutic methods through targeting free hemoglobin or heme. These biomarkers could be particularly valuable in predicting the growth rate of AAAs, where reliable markers are currently lacking. However, further extensive research is necessary to establish their potential efficacy and utility.

The present study has several strengths. Firstly, we had a relatively large sample size, which is particularly valuable given the low prevalence of AAA compared to many other CVDs^1, 2^. Additionally, the participants were consecutively recruited from a screening program^5, 25^, reducing the potential for selection bias. To ensure a comprehensive evaluation, we investigated multiple well-known markers associated with heme homeostasis in relation to both AAA prevalence and growth rate. Furthermore, our marker measurements were conducted using well-validated immunoassays, and we also employed accurate techniques like droplet digital PCR which provides absolute quantification of copy number of HP. This combination of methods allowed for robust and accurate measurement of the markers of interest. We took into account potential confounding factors, addressed missing data, and conducted sensitivity analyses to ensure the reliability and robustness of our findings. In addition, our findings were consistent with associations and correlations observed in studies on similar pathologies or in animal models of AAA. This alignment with previous findings and known biological phenomena strengthens the validity and relevance of our results.

However, it is also important to acknowledge certain limitations of the current study. This is a case-control study where sampling was done only at baseline which prevented us from inferring casual and complex-dynamic relationships between the studied markers and AAA. Additionally, our assessment of Hp phenotype and protein level relied on an indirect approach through the study of genotypes. The haptoglobin typing method developed in the current study (Hp1-1, Hp1-2, Hp2-2), does not account for the Hp0 phenotype that is a result of Hp^del^ genotype and associated with haptoglobin depletion. However, as Hp^del^ genotype has not been reported in Europe [60], it should not affect our current interpretation in an exclusively Swedish/European population. Nevertheless, modification of the method would be needed when studying non-European or mixed populations. Finally, our measurement of plasma heme cannot discriminate between cell free heme and cell-free hemoglobin. Although both the molecules, directly and indirectly [12], are associated with adverse effects, more studies are needed to account for these components separately in dynamic disease models. The present study sample was restricted to 65-year-old men, targeted by the current screening program according to established guidelines [1], AAA prevalence being low in females and younger age groups [1]. This is a common limitation of AAA studies in screening materials, and it would be valuable to test the markers identified in our study in women and other age groups to broaden the applicability of these findings.

In conclusion, the current study highlights a distinctive disruption in heme or heme-related proteins relevant for both prevalence and progression of AAA, suggesting a role for heme toxicity in AAA. It is important to note that the current findings apply to males at or over the age of 65 years who are nonetheless the most affected demographic group in AAA cases. While considering the study’s limitations, our findings underscore the potential of heme homeostasis-related markers to enhance the diagnosis and prognosis of AAA. Future longitudinal and mechanistic research studies are encouraged for validating these findings and investigating the therapeutic advantages of targeting heme toxicity in the management of AAA. Overall, the heme toxicity model could provide a promising avenue for the development of predictive markers and therapeutic targets in AAA.

### Electronic supplementary material

Below is the link to the electronic supplementary material.

Supplementary Material 1

## Data Availability

The clinical information and data used in this study cannot be made publicly available due to ethical permit restrictions. However, the analysis code and anonymized assay data that support the findings of this study are available from the corresponding author upon request.
