# Supplementary material for: Hemoglobin homeostasis in abdominal aortic aneurysm: diagnostic and prognostic potential of hemoglobin/heme and scavenger molecules
Source: BMC Cardiovasc Disord. 2024 Aug 27;24:452. doi: 10.1186/s12872-024-04131-3 (PMC11350951; doi:10.1186/s12872-024-04131-3)
Supplement: Supplementary file 1 — Supplementary Material 1 [file 12872_2024_4131_MOESM1_ESM.docx]

**Supplemental Online Content**

**Table S1.** Baseline clinical characteristics of 65-year-old men with and without abdominal aortic aneurysm (AAA) at ultrasound screening: excluding missing exposure values

**Table S2.** Correlation between biomarkers and baseline diameter

**Table S3.** Combinations of current study markers to predict AAA

**Table S4.** Correlation between current study markers with and AAA growth rate

**Table S5.** Combinations of current study marker to predict AAA growth

**Table S6.** Combinations of current study markers with previously published biomarkers to predict AAA

**Table S7**. Combinations of current study markers with previously published biomarkers to predict AAA growth

**Table S8.** Levels of biomarkers and their association with AAA risk: complete case analysis

**Table S9.** Correlation between biomarkers and baseline diameter: complete case analysis

**Table S10.** Associations between current study markers and AAA growth as continuous variable: complete case analysis

**Table S11.** Univariable and multivariable logistic regression analysis for current study markers with respect to AAA prevalence

**Table S1. Baseline clinical characteristics of 65-year-old men, with and without abdominal aortic aneurysm (AAA) at ultrasound screening: excluding missing exposure values**

| Variable | Total, n=393 | Without AAA, n=265 | With AAA, n=128 |
| --- | --- | --- | --- |
| Baseline diameter (mm), mean (SD) | 25.5 (9.9) | 19.4 (2.5) | 38.0 (7.3) |
| Growth rate (mm/year), median (IQR)  Missing, n (%) | 1.6 (2.2) | - | 1.6 (2.2)  21 (16.1) |
| Systolic BP (mmHg), mean (SD) | 144.6 (17.5) | 145.7 (17.9) | 142.4 (16.4) |
| Diastolic BP (mmHg), mean (SD) | 85.9 (9.4) | 86.0 (9.7) | 85.5 (9.0) |
| Creatinine (µmol/L), mean (SD)  Missing, n (%) | 86.8 (17.9)  2 (0.5) | 85.1 (13.5)  0 (0) | 90.3 (24.4)  2 (1.6) |
| Cholesterol (mmol/L), mean (SD)  Missing, n (%) | 5.1 (1.2)  2 (0.5) | 5.3 (1.2)  0 (0) | 4.7 (1.2)  2 (1.6) |
| Triglyceride (mmol/L), median (IQR)  Missing, n (%) | 1.5 (1.0)  14 (3.6) | 1.6 (1.0)  7 (2.6) | 1.9 (1.0)  7 (5.5) |
| HDL (mmol/L), mean (SD)  Missing, n (%) | 1.3 (0.4)  2 (0.5) | 1.4 (0.4)  0 (0) | 1.2 (0.3)  2 (1.6) |
| LDL (mmol/L), mean (SD)  Missing, n (%) | 3.3 (1.1)  11 (2.8) | 3.5 (1.0)  8 (3.0) | 3.0 (1.0)  3 (2.3) |
| Glucose (mmol/L), mean (SD)  Missing, n (%) | 6.3 (2.4)  5 (1.3) | 6.2 (2.4)  2 (0.7) | 6.5 (2.4)  3 (2.3) |
| Metformin, n (%)  Missing, n (%) | 26 (6.6)  58 (14.8) | 13 (4.9)  38 (14.3) | 13 (10.2)  20 (15.6) |
| Lipid-lowering drug use, n (%)  Missing, n (%) | 135 (34.3)  58 (14.8) | 72 (27.2)  38 (14.3) | 63 (49.2)  20 (15.6) |
| Anti-hypertensive drug use, n (%)  Missing, n (%) | 184 (46.8)  58 (14.8) | 105 (39.6)  38 (14.3) | 79 (61.7)  20 (15.6) |
| AAA family history, n (%)  Missing, n (%) | 27 (6.9)  136 (34.6) | 13 (4.9)  133 (50.2) | 14 (10.9)  3 (2.3) |
| CVD, n (%)  Missing, n (%) | 84 (21.4)  1 (0.2) | 38 (14.3)  0 (0) | 46 (35.9)  1 (0.8) |
| Type 2 diabetes, n (%)  Missing, n (%) | 29 (7.4)  1 (0.2) | 13 (4.9)  0 (0) | 16 (12.5)  1 (0.8) |
| Cancer, n (%)  Missing, n (%) | 50 (12.7)  1 (0.2) | 28 (10.6)  0 (0) | 22 (17.2)  1 (0.8) |
| Smoking ≥ 15 pack-years, n (%)  Missing, n (%) | 147 (37.4)  63 (16.0) | 69 (26.0)  35 (13.2) | 78 (60.9)  28 (21.9) |
|  |  |  |  |

*HDL: high density lipoprotein, LDL: low density lipoprotein,*

*Hb: Hemoglobin, CVD: cardiovascular disease.*

*Data are represented as mean (SD) or count (percentage).*

**Table S2. Correlation between biomarkers and baseline diameter**

|  | All | | Non-AAA | | AAA | |
| --- | --- | --- | --- | --- | --- | --- |
| Biomarker | Spearman’s rho | p-value | Spearman’s rho | p-value | Spearman’s rho | p-value |
| Hpx | 0.160 | **<0.001** | -0.063 | 0.31 | -0.133 | 0.13 |
| HO-1 | -0.210 | **<0.001** | 0.043 | 0.48 | 0.082 | 0.35 |
| Heme | 0.257 | **<0.001** | -0.110 | 0.07 | 0.098 | 0.27 |
| Hb | -0.031 | 0.53 | 0.010 | 0.87 | 0.046 | 0.59 |
| Hp phenotype* | 0.053 | 0.95 | 2.94 | 0.055 | 0.48 | 0.62 |
| Hp genotype* | 0.75 | 0.47 | 1.20 | 0.30 | 0.63 | 0.53 |

*Data presented represents F and p-values obtained from the analysis of variance (ANOVA)

Hpx: hemopexin, HO-1: heme oxygenase-1, Hb: hemoglobin, Hp: haptoglobin

**Table S3. Combinations of current study marker to predict AAA**

| Model number | Biomarkers | AUC | 95 % CI |
| --- | --- | --- | --- |
| 1 | Hpx | 0.66 | 0.60-0.72 |
| 2 | HO-1 | 0.69 | 0.63-0.74 |
| 3 | Heme | 0.71 | 0.66-0.77 |
| 4 | Hpx + HO-1 | 0.71 | 0.66-0.76 |
| 5 | Hpx + Heme | 0.74 | 0.69-0.79 |
| 6 | HO-1 + Heme | 0.76 | 0.70-0.81 |
| 7 | Hp + HO-1 + Heme | 0.76 | 0.71-0.82 |

| ROC vs ROC | p-value^*^ |
| --- | --- |
| 3 vs 1 | 0.17 |
| 3 vs 2 | 0.43 |
| 4 vs 1 | 0.06 |
| 4 vs 2 | 0.14 |
| 4 vs 3 | 0.91 |
| 5 vs 1 | **0.01** |
| 5 vs 2 | 0.14 |
| 5 vs 3 | 0.08 |
| 6 vs 1 | **0.01** |
| 6 vs 2 | **0.002** |
| 6 vs 3 | **0.007** |
| 6 vs 4 | 0.06 |
| 6 vs 5 | 0.32 |
| 7 vs 6 | 0.44 |

* Delong’s test for correlated ROC-curves

Hpx: hemopexin, HO-1: heme oxygenase-1, Hb: hemoglobin

**Table S4. Correlation between current study markers with AAA growth rate**

| Biomarker | Spearman’s rho | p-value |
| --- | --- | --- |
| Hpx | 0.160 | **<0.001** |
| HO-1 | -0.210 | **<0.001** |
| Heme | 0.257 | **<0.001** |
| Hb | -0.031 | 0.53 |
| Hp (phenotype)* | 0.26 | 0.77 |
| Hp (genotype)* | 0.83 | 0.44 |

*Data presented represents F and p-values obtained from the analysis of variance (ANOVA)

Hpx: hemopexin, HO-1: heme oxygenase-1, Hb: hemoglobin, Hp: haptoglobin

**Table S5. Combinations of current study marker to predict AAA growth**

| Model number | Biomarkers | AUC | 95 % CI |
| --- | --- | --- | --- |
| 1 | Hpx | 0.68 | 0.57-0.78 |
| 2 | Hb | 0.68 | 0.57-0.79 |
| 3 | Hpx + Hb | 0.76 | 0.67-0.86 |

| ROC vs ROC | p-value^*^ |
| --- | --- |
| 1 vs 2 | 0.96 |
| 3 vs 1 | 0.06 |
| 3 vs 2 | 0.07 |

* Delong’s test for correlated ROC-curves

**Table S6. Combinations of current study markers with previously published biomarkers to predict AAA**

| Model number | Biomarkers | AUC | 95 % CI |
| --- | --- | --- | --- |
| 1 | Hpx | 0.66 | 0.60-0.72 |
| 2 | HO-1 | 0.69 | 0.63-0.74 |
| 3 | Heme | 0.71 | 0.66-0.77 |
| 4 | Hpx + HO-1 | 0.71 | 0.66-0.76 |
| 5 | Hpx + Heme | 0.74 | 0.69-0.79 |
| 6 | HO-1 + Heme | 0.76 | 0.70-0.81 |
| 7  8 | Hpx + HO-1 + Heme  IL-6 | 0.76  0.72 | 0.71-0.82  0.67-0.78 |
| 9 | GDF_15 | 0.71 | 0.65-0.77 |
| 10 | CSTB | 0.71 | 0.64-0.77 |
| 11 | GDF-15 + CSTB | 0.76 | 0.70-0.82 |
| 12 | Heme + GDF-15 + CSTB | 0.82 | 0.77-0.87 |
| 13  14  15  16  17  18  19 | Hpx + HO-1 + Heme + GDF-15 + CSTB  Heme + IL-6  HO-1 + IL-6  Heme + HO-1 + IL-6  Heme + HO-1 + 1L-6 + GDF15  Heme + HO-1 + 1L-6 + CSTB  Heme + HO-1 + 1L-6 + GDF15 + CSTB | 0.84  0.82  0.84  0.87  0.87  0.87  0.87 | 0.79-0.89  0.77-0.86  0.79-0.88  0.83-0.91  0.82-0.92  0.82-0.92  0.82-0.92 |

| ROC vs ROC | p-value^*^ | ROC vs ROC | p-value^*^ |
| --- | --- | --- | --- |
| 3 vs 1 | 0.17 | 11 vs 3 | 0.38 |
| 3 vs 2 | 0.43 | 12 vs 3 | **<0.001** |
| 4 vs 1 | 0.06 | 11 vs 6 | **0.02** |
| 4 vs 2 | 0.14 | 11 vs 10 | **0.03** |
| 5 vs 1 | **0.01** | 12 vs 11 | **0.006** |
| 5 vs 3 | 0.08 | 8 vs 11 | 0.35 |
| 6 vs 2 | **0.002** | 8 vs 14 | **<0.001** |
| 6 vs 3 | **0.007** | 16 vs 11 | **0.01** |
| 6 vs 4 | 0.06 | 16 vs 14 | **0.002** |
| 6 vs 5 | 0.32 | 16 vs 15 | **0.008** |
| 7 vs 6 | 0.44 | 16 vs 17 | 0.15 |
| 11 vs 9 | **0.02** | 16 vs 18 | 0.20 |
| 11 vs 10 | **0.03** | 16 vs 19 | 0.07 |

* Delong’s test for correlated ROC-curves

Hpx: hemopexin, HO-1: heme oxygenase-1, GDF-15: Growth differentiation factor-15; CSTB: Cystatin B; IL-6: Interleukin-6

**Table S7. Combinations of current study markers with previously published biomarker to predict AAA growth**

| Model number | Biomarkers | AUC | 95 % CI |
| --- | --- | --- | --- |
| 1 | Hpx | 0.68 | 0.57-0.78 |
| 2 | Hb | 0.68 | 0.57-0.79 |
| 3 | Hpx + Hb | 0.76 | 0.67-0.86 |
| 4 | MPO | 0.68 | 0.58-0.78 |
| 5 | MPO + Hpx | 0.70 | 0.60-0.81 |
| 6 | MPO + Hb | 0.76 | 0.67-0.85 |
| 7 | MPO + Hpx + Hb | 0.78 | 0.69-0.87 |
| 8 | IL-6 | 0.50 | 0.39-0.62 |
| 9 | IL-6 + Hpx | 0.71 | 0.61-0.81 |
| 10 | IL-6 + Hb | 0.71 | 0.60-0.83 |
| 11 | IL-6 + Hpx + Hb | 0.79 | 0.70-0.89 |
| 12 | IL-6 + MPO + Hpx + Hb | 0.80 | 0.71-0.90 |

| ROC vs ROC | p-value^*^ |
| --- | --- |
| 1 vs 2 | 0.96 |
| 3 vs 1 | 0.06 |
| 3 vs 2 | 0.07 |
| 4 vs 1 | 0.91 |
| 4 vs 2 | 0.90 |
| 4 vs 3 | 0.22 |
| 4 vs 5 | 0.62 |
| 4 vs 6 | 0.10 |
| 4 vs 7 | 0.06 |
| 7 vs 3 | 0.62 |
| 9 vs 11  10 vs 11 | 0.08  0.06 |
| 11 vs 3 | 0.23 |
| 11 vs 7 | 0.55 |
| 11 vs 12 | 0.96 |

* Delong’s test for correlated ROC-curves

Hpx: hemopexin, Hb: hemoglobin, MPO: myeloperoxidase, IL-6: interleukin-6

**Table S8. Levels of biomarkers and their association with AAA risk: CCA**

| Marker | Non-AAA | AAA | p-value | Adjusted p-value^1^ | OR^2^ | 95% CI^2^ | AUC | 95% CI | p-value |
| --- | --- | --- | --- | --- | --- | --- | --- | --- | --- |
| Hpx (mg/mL), mean (SD)  Missing, n (%) | 1.56 (1.0)  10 (3.6) | 1.93 (1.08)  12 (8.4) | **<0.001** | **<0.001** | 1.41 | 1.14-1.76 | 0.65 | 0.59-0.71 | **<0.001** |
| HO-1 (ng/mL), mean (SD)  Missing, n (%) | 3.70 (1.5)  10 (3.6) | 2.95 (1.45)  12 (8.4) | **<0.001** | **<0.001** | 0.53 | 0.39-0.70 | 0.68 | 0.62-0.74 | **<0.001** |
| Heme (µM), mean (SD)  Missing, n (%) | 23.2 (10.3)  10 (3.6) | 38.2 (24.3)  12 (8.4) | **<0.001** | **<0.001** | 2.49 | 1.92-3.31 | 0.71 | 0.66-0.77 | **<0.001** |
| Hb (g/L), mean (SD)  Missing, n (%) | 147.9 (10.2)  9 (3.2) | 146.9 (11.5)  6 (4.2) | 0.39 | 0.23 | 0.91 | 0.74-1.12 | 0.53 | 0.47-0.59 | 0.15 |
| Hp type, n (%)  Hp1-1  Hp2-1  Hp2-2  Missing, n (%) | 50 (17.9)  130 (46.6)  93 (33.3)  6 (2.1) | 23 (16.2)  63 (44.4)  50 (35.2)  6 (4.2) | 0.85 | 0.84 | 0.95  Ref  1.11 | 0.53-1.68  -  0.70-1.75 | 0.52 | 0.46-0.57 | 0.28 |
| Hp genotype, n (%)  GG  GA  AA  Missing, n (%) | 171 (61.3)  92 (33.0)  10 (3.6)  6 (2.1) | 79 (55.6)  49 (34.5)  8 (5.6)  6 (4.2) | 0.48 | 0.80 | Ref  1.15  1.73 | -  0.74-1.78  0.64-4.56 | 0.53 | 0.47-0.58 | 0.16 |

^1^ Adjusted for smoking ≥ 15 pack-years, CVD and medication (hypertension, lipid, metformin).

^2^ Odds ratios for standardized (continuous) biomarkers (mean = 0 and SD = 1).

CCA: complete case analysis, Hpx: hemopexin, HO-1: heme oxygenase-1, Hb: hemoglobin, Hp: haptoglobin

**Table S9. Correlation between biomarkers and baseline diameter: CCA**

|  | All | | Non-AAA | | AAA | |
| --- | --- | --- | --- | --- | --- | --- |
| Biomarker | Spearman’s rho | p-value | Spearman’s rho | p-value | Spearman’s rho | p-value |
| Hpx | 0.160 | **0.002** | -0.059 | 0.34 | -0.152 | 0.08 |
| HO-1 | -0.211 | **<0.001** | 0.039 | 0.52 | 0.066 | 0.45 |
| Heme | 0.250 | **<0.001** | -0.092 | 0.13 | 0.098 | 0.27 |
| Hb | -0.034 | 0.50 | 0.006 | 0.93 | 0.046 | 0.59 |
| Hp (phenotype)* | 0.028 | 0.97 | 3.035 | 0.05 | 0.274 | 0.76 |
| Hp (genotype)* | 0.82 | 0.44 | 1.24 | 0.30 | 0.55 | 0.57 |

*Data presented represents F and p-values obtained from the analysis of variance (ANOVA)

CCA: complete case analysis, Hpx: hemopexin, HO-1: heme oxygenase-1, Hb: hemoglobin, Hp: haptoglobin

**Table S10. Associations between biomarkers and AAA growth as continuous variable: CCA**

| Biomarker | β | p-value | Adjusted β^1^ | p-value | AUC^2^ | 95% CI |
| --- | --- | --- | --- | --- | --- | --- |
| Hpx | -0.20 | **<0.001** | -0.14 | **0.009** | 0.68 | 0.58-0.78 |
| HO -1 | 0.13 | 0.18 | 0.00 | 0.99 | 0.57 | 0.46-0.68 |
| Heme | 0.07 | 0.14 | 0.06 | 0.22 | 0.70 | 0.60-0.80 |
| Hb | 0.16 | **0.002** | 0.13 | **0.02** | 0.68 | 0.57-0.79 |
| Hp (phenotype)  Hp1-1  Hp2-1  Hp2-2 | 0.10  Ref  -0.01 | 0.53  -  0.92 | 0.16  Ref  0.06 | 0.30  -  0.61 | 0.56 | 0.46-0.67 |
| Hp (genotype)  GG  GA  AA | Ref  -0.03  0.07 | -  0.83  0.74 | Ref  -0.01  -0.06 | -  0.92  0.80 | 0.52 | 0.42-0.62 |

^1^ Adjusted for baseline diameter and metformin

^2^AUC calculated using logistic regression with *fast- or slow- growing AAA* as outcome.

CCA: complete case analysis, Hpx: hemopexin, HO-1: heme oxygenase-1, Hb: hemoglobin, Hp: haptoglobin

**Table S11. Odds ratio and 95% confidence interval for AAA prevalence by current study markers**

| Marker | Crude | | | Adjusted^a^ | | | Adjusted^b^ | | |
| --- | --- | --- | --- | --- | --- | --- | --- | --- | --- |
|  | OR | 95% CI | p-value | OR | 95% CI | p-value | OR | 95% CI | p-value |
| Hpx | 1.42 | 1.61, 1.77 | 0.001 | 1.49 | 1.17, 1.93 | 0.002 | 1.73 | 1.24, 2.46 | 0.002 |
| HO-1 | 0.51 | 0.38, 0.66 | <0.001 | 0.56 | 0.41, 0.75 | <0.001 | 0.25 | 0.13, 0.42 | <0.001 |
| Heme | 2.48 | 1.92, 3.28 | <0.001 | 2.34 | 1.74, 3.27 | <0.001 | 2.70 | 1.81, 4.18 | <0.001 |
| Hb | 0.90 | 0.73, 1.10 | 0.29 | 0.94 | 0.74, 1.19 | 0.60 | 0.94 | 0.68, 1.28 | 0.68 |
| Hp type |  |  |  |  |  |  |  |  |  |
| Hp1-1 | Ref |  |  | Ref |  |  | Ref |  |  |
| Hp2-1 | 0.92 | 0.51, 1.62 | 0.78 | 1.32 | 0.67, 2.61 | 0.41 | 0.61 | 0.25, 1.42 | 0.26 |
| Hp2-2 | 1.10 | 0.70, 1.72 | 0.69 | 1.05 | 0.61, 1.79 | 0.87 | 0.65 | 0.31, 1.30 | 0.23 |
| Hp genotype |  |  |  |  |  |  |  |  |  |
| GG | Ref |  |  | Ref |  |  | Ref |  |  |
| GA | 1.11 | 0.72, 1.71 | 0.64 | 0.89 | 0.53, 1.49 | 0.67 | 0.86 | 0.45, 1.48 | 0.52 |
| AA | 1.67 | 0.62, 4.37 | 0.30 | 0.81 | 0.26, 2.47 | 0.71 | 0.32 | 0.04, 1.77 | 0.22 |

*Hpx: Hemopexin; HO-1: Heme-oxygenase-1; Hb: Hemoglobin; Hp:Haptoglobin*

*^a^ model a: Adjusted for smoking ≥ 15 pack-years, CVD, and medication (hypertension, lipid, metformin).*

^b^ *model b: Adjusted for smoking ≥ 15 pack-years, cancer, low density lipoprotein (LDL), triglyceride (TG) and interleukin 6 (IL-6).*

*Marker values are standardized (mean = 0 and SD = 1).*
